# Temporal but Not Spatial Variability during Gait Is Reduced after Selective Dorsal Rhizotomy in Children with Cerebral Palsy

**DOI:** 10.1371/journal.pone.0069500

**Published:** 2013-07-26

**Authors:** Mustafa Sinan Bakir, Franziska Gruschke, William R. Taylor, Ernst Johannes Haberl, Ilya Sharankou, Carsten Perka, Julia F. Funk

**Affiliations:** 1 Center for Musculoskeletal Surgery, Department of Orthopaedics, Charité–Universitätsmedizin Berlin, Berlin, Germany; 2 Center for Musculoskeletal Surgery, Julius Wolff Institute, Center for Sports Science and Sports Medicine Berlin, Charité–Universitätsmedizin Berlin, Berlin, Germany; 3 Institute for Biomechanics, ETH Zürich, Zürich, Switzerland; 4 Department of Paediatric Neurosurgery, Charité–Universitätsmedizin Berlin, Berlin, Germany; 5 Social Paediatric Center, Charité–Universitätsmedizin Berlin, Berlin, Germany; Katholieke Universiteit Leuven, Belgium

## Abstract

**Introduction:**

Variability in task output is a ubiquitous characteristic that results from non-continuous motor neuron firing during muscular force generation. However, variability can also be attributed to errors in control and coordination of the motor neurons themselves in diseases such as cerebral palsy (CP). Selective dorsal rhizotomy (SDR), a neurosurgical approach to sever sensory nerve roots, is thought to decrease redundant or excessive afferent signalling to intramedullary neurons. In addition to its demonstrated ability to reduce muscular spasticity, we hypothesised that SDR is able to decrease variability during gait, the most frequent functional motor activity of daily living.

**Methods:**

Twelve CP children (aged 6.1±1.3yrs), who underwent SDR and performed gait analysis pre- and 12 months postoperatively, were compared to a control group of eleven typically developing (TD) children. Coefficients of variability as well as mean values were analysed for: temporal variables of gait, spatial parameters and velocity.

**Results:**

Gait parameters of cadence (*p = 0.006*) and foot progression angle at mid-stance (*p = 0.041*) changed significantly from pre- to post-SDR. The variability of every temporal parameter was significantly reduced after SDR (*p = 0.003–0.049*), while it remained generally unchanged for the spatial parameters. Only a small change in gait velocity was observed, but variability in cadence was significantly reduced after SDR (*p = 0.015*). Almost all parameters changed with a tendency towards normal, but differences between TD and CP children remained in all parameters.

**Discussion:**

The results confirm that SDR improves functional gait performance in children with CP. However, almost exclusively, parameters of temporal variability were significantly improved, leading to the conjecture that temporal variability and spatial variability may be governed independently by the motor cortex. As a result, temporal parameters of task performance may be more vulnerable to disruption, but also more responsive to treatment success of interventions such as SDR.

## Introduction

Cerebral palsy (CP) is defined as a symptom complex that originates from a non-progressive disorder of the immature brain [1] including various types and expressions of persistent motor function alterations. The bilateral spastic subtype is the most common manifestation of CP [2], which affects motion patterns and muscular control. The exact cause of CP associated spasticity remains unclear, but one plausible explanation is excessive afferent inputs onto the intramedullary neurons [3]. Physiologically any excessive incoming excitatory signals are filtered by inhibitory signals from the corticospinal tract, but this regulatory mechanism is disrupted in the case of CP associated cerebral damage [4] leading to spasticity and uncontrolled movements. It is therefore reasonable that reorganisation of a CP child’s incoming neuromotor signalling could alter and possibly reduce abnormal motion patterns [5].

Selective dorsal rhizotomy (SDR) is a well-known and frequently utilized therapeutic option for reducing spasticity in children with CP [6], [7]. Although the exact mechanisms behind its success remain hidden, it has been a common assumption that SDR reduces muscular spasticity by eliminating redundant or excessive afferent signalling, thereby leading to more balanced central processing. Additional benefits are thought to include an enhancement of motor function [7]–[9] and even gait performance [10], [11], and can thus help lead to improved participation in social life and peer group activities [12], [13].

While mean summary measures of gait such as joint angles and stride length have proved useful for assessing movement ability in children with CP [14], variability of motor patterns has recently come to light as a clear measure of task performance [15]–[17]. Variability is the natural difference that occurs between repetitions of movement strides or gait, and, although opinions differ as to the causes of motor variability [18], [19], it is generally thought to result from neuromuscular noise in the sensori-motor system [20]–[22], where the firing of motor neurons generates a non-continuous muscular force output. However, kinematic or kinetic variability can also be generated by errors or deficits in the motor control system [17], [23]. As a consequence, variability of movement trajectories is thought to be a key parameter of dynamic instability during walking [15], [23]–[25], and could thus provide an effective measure of motor ability, including control [26] and coordination of the lower limb musculature, as well as a tool for assessing progressive changes in disease status or the efficacy of therapeutic interventions.

It is entirely plausible that deteriorated gait patterns [27], [28] and spatio-temporal variability during walking in children with CP could be related to ineffective noise regulation [29] that might be improved after SDR therapy. While the influence of CP on gait patterns has been well reported in the literature, including the effect of different treatments [30], as well as the impact of CP on gait variability [26]–[28], [31], changes in gait variability that occur after SDR have not yet been investigated. Since it is conceivable that disturbances in the motor system of children with CP result from spasticity [27] and that the output variability is a product of input disturbances [32], we hypothesised that reducing the volume of afferent input through SDR might be an effective treatment not only to reduce spasticity but also to reduce variability during walking. The aim of this investigation was therefore to evaluate the effect of SDR on gait variability in children with CP.

## Materials and Methods

### Patient Population and Selection Criteria

Thirteen children diagnosed with CP, were examined before and 12 months after undergoing SDR (Table 1). One child, who presented a change in gait status from assisted to postoperative unassisted walking was excluded due to the known influence on parameters of gait variability [33], [34]. As a result, twelve children (5 female, 7 male) with a mean age of 6.1 (±1.3, range from 4.4 to 8.5) years, were analysed in this study. The parents of all children gave their written, informed consent (including data publication and video recording), to participate in this study, which was approved by the local ethics committee.

**Table 1:** Participants’ demographic, anthropometric and clinical data.

	TDC	SDR pre	SDR post
**Number of patients**	11	12	12
**Age [yrs]**	6.5 (±1.9)	6.1 (±1.3)	7.1 (±1.3)
**Gender (m/f)**	5/6	7/5	7/5
**Height [cm]**	122.6 (±13.1)	116.2 (±9.4)	123.3 (±8.9)
**Weight [kg]**	22.6 (±5.7)	19.9 (±3.1)	22.3 (±3.3)
**Leg length [cm]**	62.6 (±10.1)	57.4 (±4.2)	62.0 (±4.8)
**GMFCS**	–	5×I, 7×II	5×I, 7×II
**GMFM**	–	89.7 (±5.3)	94.2 (±4.2)
**MAS**	0 (±0)	1.5 (±0.4)	0.6 (±0.5)
**Strength**	5.0 (±0)	3.5 (±0.3)	3.9 (±0.4)
**Number of strides analysed**	66.0 (±29.2)	79.1 (±42.5)	49.5 (±12.7)

Legend: Due to normally distributed values, all parameters were presented as a mean (±standard deviation); pre = preoperative, post = 12 months postoperative, TDC = typically developing children; yrs = years, m = male, f = female; GMFCS = Gross Motor Function Classification System; GMFM = Gross Motor Function Measure; MAS = Modified Ashworth Scale; Strength: measured on a 5 point scale.

Since SDR is non-reversible, strict and consistent selection criteria according to Peacock et al. [35] were applied for all children to be considered for the therapy. For inclusion, predominantly spastic bilateral CP with Gross Motor Function Classification System (GMFCS) level I to level III [36], the absence of contractures, a good compliance and high motivation, as well as an age between three and ten years at time of surgery, were all necessary. Children without a minimum degree of forward locomotion or with previous multilevel surgery were excluded, together with children with other movement disorders (e.g. athetosis), structural orthopaedic deformities or multiple contractures.

The clinical examination algorithm also included the gross motor function measure (GMFM-88) [37], spasticity evaluation via the modified Ashworth scale (MAS) [38], and a measurement of muscle strength on a 5 point scale according to Daniels and Worthingham [39]. Furthermore, children and parents were asked regarding the success of the surgery and if they were willing to undergo this procedure again.

In addition, an age-matched control group consisted of eleven (6 female, 5 male) typically developing (TD) children who had a mean age of 6.5 (±1.9, range 4.0 to 9.3) years at the time of gait analysis (Table 1), and are presented as reference values in order to establish the magnitude of any pre- to post-operative changes in parameters of the children with CP.

### Operation Procedure and Postoperative Treatment

SDR was performed by a single surgeon via single-level laminectomy at the level of the medullary conus to ensure minimal risk of progressive lumbar instability [40]. About 50% of the afferent nerves of level L1 to S1 were severed, where those with the most pathological response during intraoperative neurophysiological testing with electric impulse stimulation and EMG measurement were selected for cutting. Postoperatively the children underwent a three week intensive subject-specific rehabilitation program focussing on training and strengthening. After leaving the hospital, all children were encouraged to continue with out-patient rehabilitation for at least 3 further months at their primary care physical therapists, with the recommendation to focus on gait training.

### Equipment

**Figure 1:**
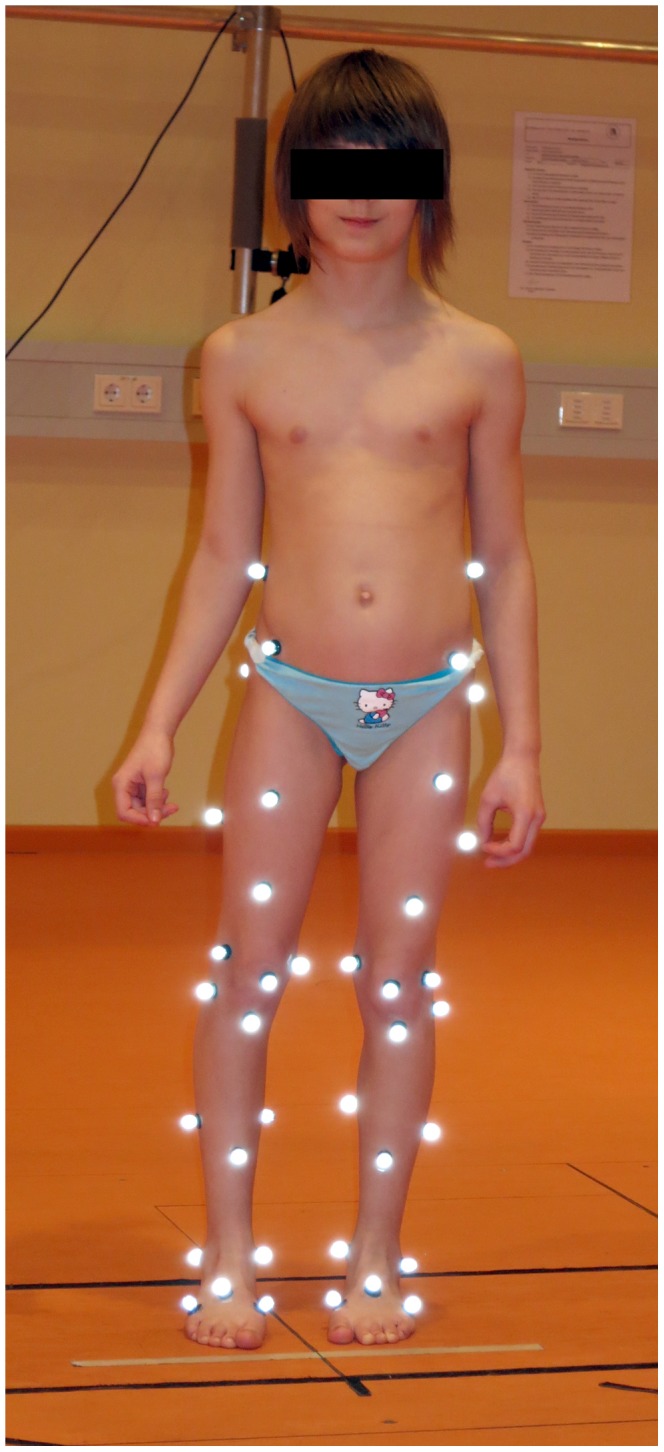
Marker set used throughout gait analyses. Written, informed permission was expressly provided by the legal guardian of the child shown to publication of their photograph, as outlined in the PLOS consent form.

Instrumented gait analysis was performed using a 10 camera three-dimensional motion capture system (Vicon, OMG, UK) at 120 Hz. A set of lower limb reflective markers (diameter 14 mm) was attached to the participant’s skin by an experienced observer according to our standard protocol (Figure 1) [41], with slight modifications for an improved matching to the children’s spastic constitution. For the assessment of ground reaction forces, two tri-axial force plates (AMTI OR6-7-1000, Watertown, Massachusetts, USA) recording at 960 Hz were used.

### Gait Protocol

All children walked barefoot on a 10 m straight and level walkway at self-selected and preferably constant speed. They were allowed to use their assistive devices or hold someone’s hand if necessary to complete the assessment. For familiarisation with the gait laboratory the children walked a few practice laps. To minimize acceleration and deceleration caused by repetitive stop and go movements, toy targets were used as turning pylons and were placed beyond the end of the walkway. Sufficient walks were recorded to allow at least eight full trials to be analysed, where a trial was considered at least two full gait cycles (strides) of each leg. Trials were excluded from analysis when the child was distracted or stopped during walking, and videos from the side and from the front/back were recorded for visual confirmation of data coherence.

### Data Analysis

After labelling, the first and last three seconds of each trial were removed to avoid perturbations during movement initiation and termination. Spatio-temporal parameters of gait were then calculated from the foot marker kinematic data. Heel-strike (or initial contact in the case of toe-first contact in the CP children) and toe-off were detected via a foot velocity algorithm [42], using the heel markers of each foot. Using these two events, the parameters of stride, step, stance and swing phases, and double limb support were computed [43]. Mid-swing time was defined as the time at which mid-swing occurred, and is presented as a percentage of the total swing phase where the distance between the left and right medial malleoli markers was at a minimum. A stride was defined as two consecutive initial contacts of the same foot. The difference in the forward displacement of the right and the left heel markers at each consecutive initial contact was used to determine the step length. Stride height was computed as the maximum value of the vertical component of the heel marker. Finally, the direction of walking progression was determined as the line from the heel marker at heel strike to its position at the following heel strike, in order to provide a reference for calculation of the foot progression angle (FPA). Here, the direction of the foot was defined as the vector from the heel marker to the mid-point of the Os Metatarsale I and V markers. The FPA was then considered as the angle between the walking progression line and the foot direction at the time of the mid-stance event. A positive FPA represented toe-out.

All spatial parameters were converted into dimensionless units by normalising for leg length because of significant anthropometric differences between preoperative and follow-up measurements (p<0.001) due to considerable growth of the children [44].

### Analysis of Variability

In addition to velocity (walking speed), parameters of gait variability were calculated from the kinematic data including temporal parameters: cadence, stride time, stance time, swing time, double limb support time and mid-swing time; and spatial parameters: stride and step length, stride height, and FPA. Stride-to-stride variability was determined using the coefficient of variation (CV), where CV = (standard deviation/mean) ×100. The CV of each parameter was calculated for each child using the mean of both legs averaged over all evaluated gait cycles, with low values representing low amounts of variability between gait cycle repetitions for each given parameter. For determination of the CV of the FPA unsigned values were used in order to ensure a positive CV and thus provide the basis for improved comparison.

### Statistics

Statistical analysis was performed using the SPSS software (v20, IBM, Champaign, IL). All spatio-temporal parameters were expressed in absolute normalised values or in relation to the gait cycle. Due to normally distributed values (tested using the Kolmogorov-Smirnov-test), all anthropometric and clinical parameters were tested using the paired t-test (Table 1). However, due to partially non-normally distributed changes in gait parameters after SDR, the paired Wilcoxon signed-rank test was used in these cases. The level of significance was set at p<0.05.

## Results

The clinical parameters for assessing function, spasticity and strength changed as follows: function measured with the GMFM-88 increased significantly (p = 0.001) from 89.7±5.3 to 94.2±4.2, 12 months after SDR. Spasticity measured using the MAS decreased significantly (p<0.001) from 1.5±0.4 to 0.6±0.5. The overall muscle strength of the lower limbs increased slightly but statistically significantly (p<0.001) from 3.5±0.3 to 3.9±0.4. Improvement with regard to performing sports and activities of daily living was reported by all children and/or parents, which was the key reason underlying the subjective confirmation of the SDR procedure success.

The absolute walking velocity of the CP children increased from 0.80 m/s preoperatively to 0.93 m/s twelve months postoperatively, which was still slower than that of the age matched TD children (1.14 m/s). In addition to this increase in speed from pre- to post-operative, the children with CP showed a larger step length (41.2 cm preoperative vs. 48.1 cm postoperative; 48.7 cm in the controls) and an increase in leg length of 2.6 cm (pre-OP 57.4 cm, post-OP 62.0 cm) at the 12 months follow-up period (Table 1). The cadence of the CP children, on the other hand, was significantly reduced after SDR (p = 0.006) (Table 2). The normalised velocity of the CP children increased from 73% to 83% of the TD children’s velocity after SDR therapy, but this was not significant. The FPA changed significantly from an internal rotation of 8.6° prior to SDR to an external rotation of 2.6° twelve months post SDR (Table 2).

**Table 2:** Spatio-temporal parameters of kinematics, shown preoperatively and 12 months postoperatively.

		TDC	SDR pre	SDR post	p-value
**Temporal**	**Cadence** [steps/s]	2.23 (2.12/2.43)	2.46 (2.17/2.60)	2.17 (2.03/2.27)	**0.006** *
	**Stride time** [norm]	3.45 (3.21/3.86)	3.45 (3.15/4.02)	3.76 (3.48/3.84)	0.084
	**Stance phase** [%]	60.1 (59.1/60.4)	61.3 (59.9/65.3)	60.8 (59.4/62.4)	0.480
	**Swing phase** [%]	39.9 (39.6/40.9)	38.7 (34.7/40.1)	39.2 (37.6/40.6)	0.480
	**Double limb support** [%]	10.1 (9.0/10.7)	11.4 (10.0/15.5)	10.8 (9.5/12.0)	0.239
	**Mid-swing time** [%]	43.7 (43.4/46.4)	43.8 (40.8/45.7)	45.9 (43.5/47.2)	0.182
**Spatial**	**Stride length** [norm]	1.55 (1.41/1.63)	1.10 (1.06/1.36)	1.40 (1.11/1.52)	0.084
	**Stride height** [norm]	0.32 (0.30/0.33)	0.30 (0.29/0.31)	0.31 (0.29/0.33)	0.060
	**Step length** [norm]	0.79 (0.72/0.82)	0.71 (0.66/0.75)	0.79 (0.70/0.82)	0.272
	**FPA at mid-stance** [°]	17.2 (12.4/21.8)	−8.6 (−15.4/3.3)	2.6 (−10.2/9.0)	**0.041** *
**Spatio-temporal**	**Velocity** [norm]	0.45 (0.41/0.51)	0.33 (0.28/0.43)	0.38 (0.29/0.45)	0.480

Legend: Due to partially non-normally distributed values, all general spatio-temporal parameters concerning SDR related changes are presented as a median together with interquartile range; TDC = typically developing children, pre = preoperative, post = 12 months postoperative; [%] = percentage of stride time or percentage of related gait cycle phase (swing phase), [°] = degree; [norm] = normalised; FPA = Foot progression angle: positive values represent externally rotated feet;

* = significance with p<0.05 between pre and post SDR.

**Figure 2:**
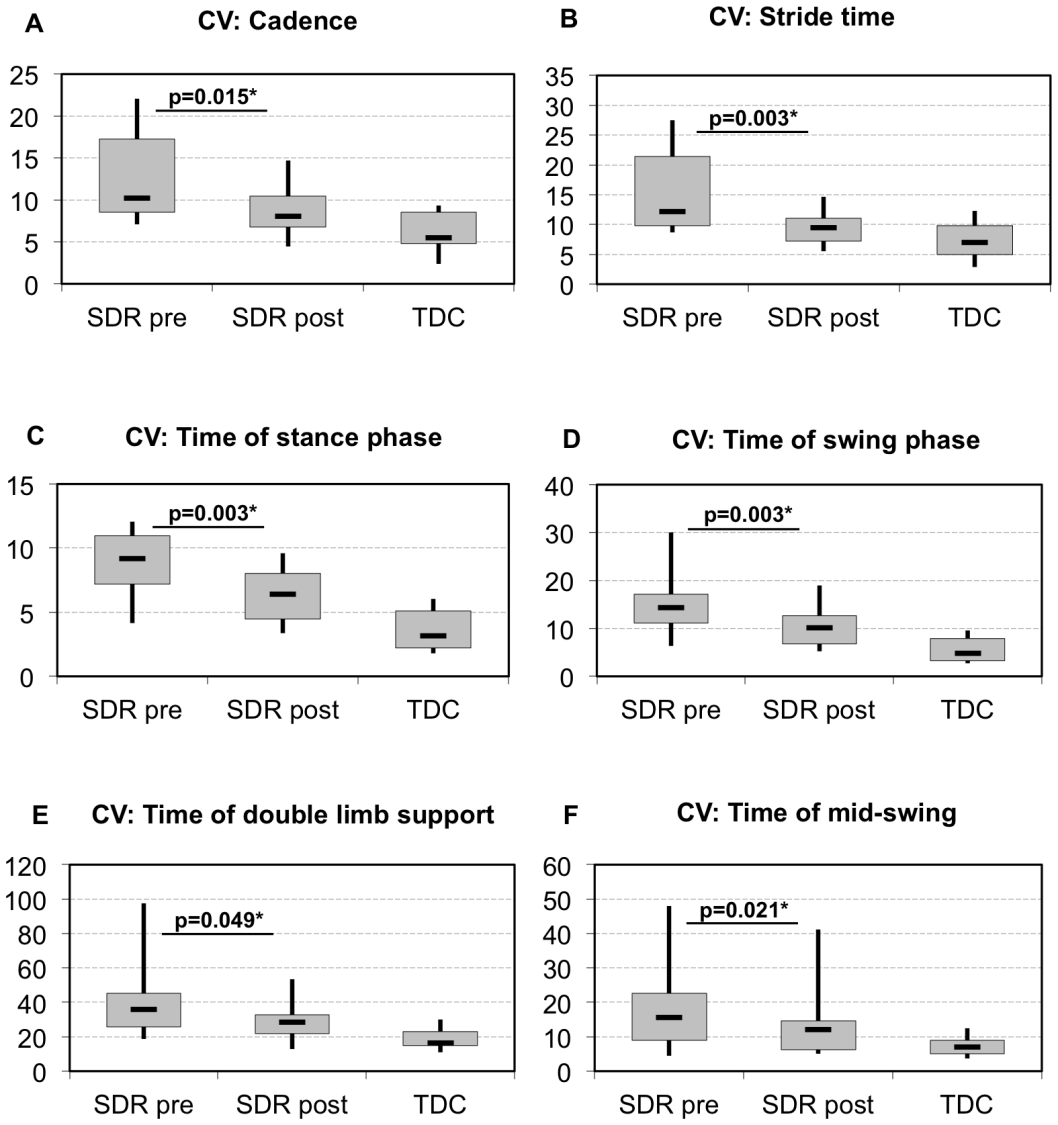
CV of temporal parameters pre- and post-SDR compared to typically developing children (TDC). Levels of significance are shown for the children with CP between the preoperative and 12 months post-SDR therapy time points. All values are presented as medians, shown together with interquartile ranges as well as minimum and maximum values.

**Figure 3:**
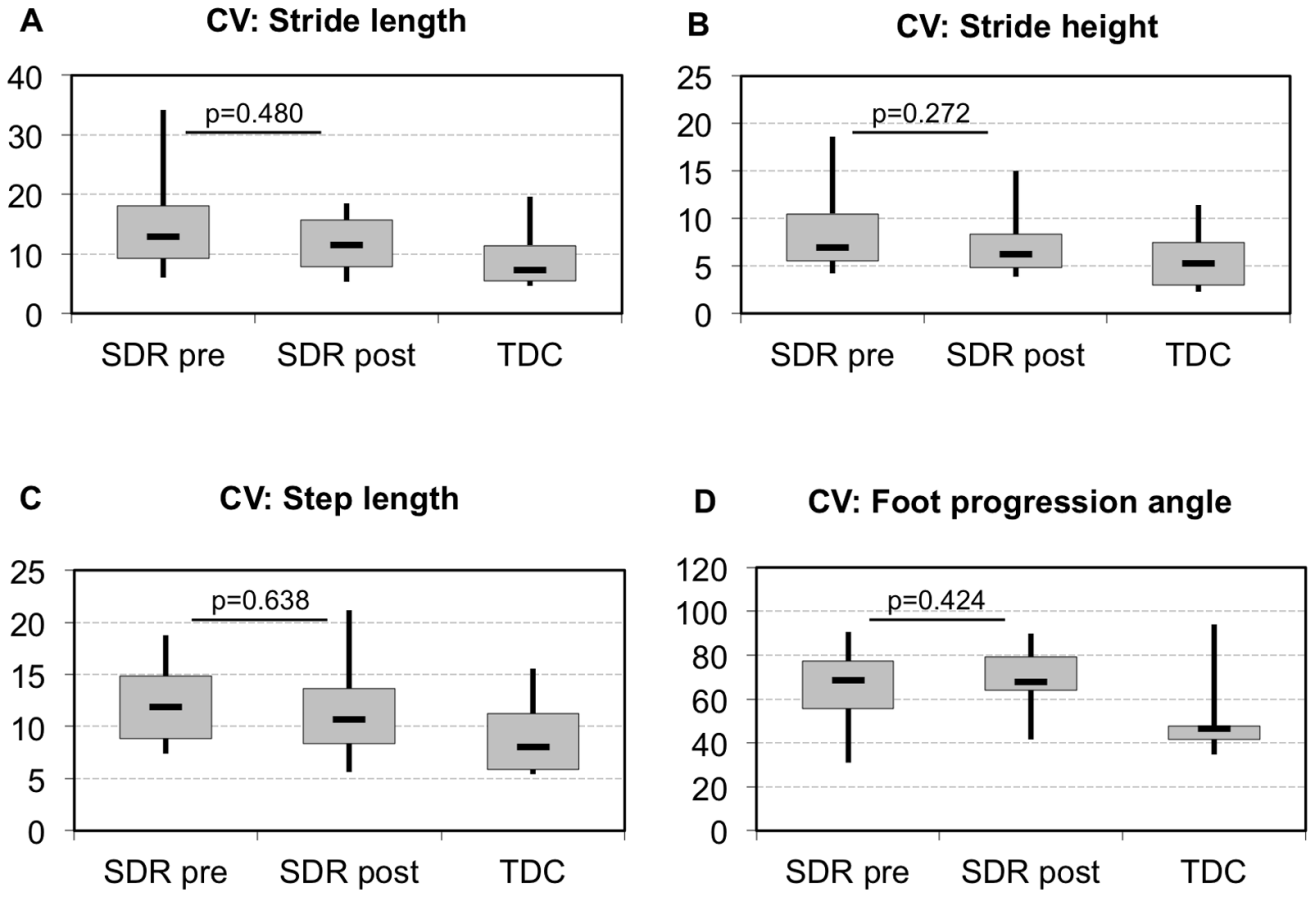
CV of spatial parameters pre- and post-SDR compared to typically developing children (TDC). Levels of significance are shown for the CP children between the preoperative and 12 months post-SDR therapy time points. All values are presented as medians, shown together with interquartile ranges as well as minimum and maximum values.

The coefficient of variability was reduced in all spatio-temporal parameters of the CP children’s kinematics after SDR, but the levels of variability of the TD children were never reached. Although a tendency towards the variability values of the TD children was observed for all parameters, a significant reduction was found only for the CV of temporal parameters (cadence, stride time, stance phase, swing phase, double limb support and time of mid-swing) (Figure 2). No significant change was observed in any spatial parameter of variability (Figure 3).

## Discussion

Reduced muscular control is a major limitation in children with CP. While evaluations of their absolute movement patterns can help in unravelling the degree of skeletal deformity or spasticity, as well as functional deficits, these measures do not necessarily reveal the level of motor control, partially due to progressive adaptation of the musculoskeletal system. An examination of the variability of repetitive tasks, on the other hand, can provide an insight into the control of the muscles, and therefore allows a critical assessment of the functional benefits to be gained from specific therapies. In this study, we observed that functional motor task performance, but most importantly only temporal and not spatial parameters of gait variability, were significantly improved in children with CP one year after selective dorsal rhizotomy. While additional research that includes the use of e.g. kinetics, EMG and even theta burst stimulation approaches could help to elucidate the central control mechanisms that govern temporal and spatial differences in motor function, the implications of these findings are that temporal (i.e. rhythmicity) and spatial aspects of movement might be governed independently within the motor cortex. As a result, temporal parameters of task performance may be more vulnerable to disruption, but also more responsive to treatment success using interventions such as SDR.

Since muscles are the actuators of force and movement in the human body, and are driven by non-continuous firing of multiple motor neurons, gait patterns even in entirely healthy subjects will always show a periodicity that exhibits small differences from one step to the next [17]. Additional variability arises from the quality of motor command [17], [23], where insufficient dynamic error correction of neuromuscular noise during task performance is known to lead to increased variability [15]. Determination of the levels of variability between task repetitions is therefore a useful tool for accessing the levels of neuromuscular noise and control, and therefore task performance [15]–[17], as opposed to the actual magnitude of the signal (i.e. absolute movement). In this study, the capture of over 30 free-walking steps [45] for each child (Table 1) has been used to assess gait variability, but additional research is clearly required to explore the reliability of variability in this cohort of young children with motor function alterations. The CV was then chosen for examination in this analysis as a meaningful measure of variability that allows comparisons to be performed even in cases of large differences in group means [46]. Until now, no data exist concerning gait variability as a measure of functional task performance after SDR, although the impact of CP on task variability has been characterised using the CV of step length and stride time [28]. Interestingly a significantly higher CV of the temporal parameters cadence and single support time has been observed in early walkers with CP compared to TD children while the CV of step length and velocity was not significantly different between the groups [26]. This study presents a similar tendency of unequally affected spatial and temporal parameters. Unfortunately, the assessment of muscle activity using e.g. EMG was not possible within this study, and such approaches are considered indispensable for understanding spastic muscle activity. From a movement and neuromuscular control perspective, however, our results confirm that variability of motor task performance is greater in patients with neuromuscular disorders [15]–[17], [47], even though the consequences of this increase are sometimes unclear.

Similar to other studies investigating SDR that assessed children of comparable age and motor function score [5], [48], [49], our prospective study examined a relatively small population of patients, exacerbated by the exclusion of one child due to a change in gait status. Importantly, this child displayed a considerable improvement in gait ability from assisted to independent ambulation post-operatively, thus ensuring a conservative outlook on the remaining results. Furthermore, while the very strict selection criteria applied for the SDR surgery [35] may potentially limit the widespread interpretation of our results, we were able to achieve a homogeneous patient cohort. As a result, the partially non-normal distributions observed for some parameters could indicate a range of outcome success in the children, with some of them gaining a greater benefit than others. While this result remains to be confirmed in studies with larger cohorts, it might suggest that CP children who will gain a large benefit from SDR therapy could be better identified prior to surgery.

To our knowledge, only limited possibilities exist to improve motor control in persons with neuromuscular disorders [50]. SDR is a therapy that specifically modifies and limits signalling input to the motor control system. Its impact on the variability of motor task performance has been clearly demonstrated in this study, suggesting an improvement to the neuromotor control of the muscles. The observed mean parameters of walking velocity (0.80 m/s), absolute step and stride length of 0.41 m and 0.66 m respectively and longer stance phase of 61.3% and double limb support phase of 11.4% observed in our study are comparable to data of other study groups working with CP children [26], [51], [52], but showed no significant differences from pre- to post-operative. The clinical parameters on the other hand showed a significant improvement one year after SDR, with children displaying increased function and strength, as well as reduced spasticity, which is also consistent with previous research [6], [7], [53]. In a similar manner, the spatio-temporal parameters evaluated in this study were comparable to previously published outcomes after SDR [7], [9], [53], although patient selection, surgical technique, and gait analysis technique were somewhat variable [7].

For the first time, however, this study presents data that temporal but not spatial parameters of gait variability can be significantly decreased in children with CP twelve months after SDR. The resulting decreased variability of motor task performance after SDR may lead to higher motor skills. Interestingly, these observations are consistent with the patients’ own subjective impressions that they were more functional and able to undertake more activities of daily living (e.g. playing sports, riding a bicycle, etc.). Although the role of age (i.e. 12 months older at the follow-up time point) cannot be excluded [54], the task performance changed with a clear tendency towards a more normal distribution, even though differences between TD and CP children still remained one year after SDR.

FPA was the only spatial parameter to show significance between the pre- and the post-operative conditions. We assume that the 11.2° change in FPA from inwardly rotated to the more normal outwardly tended feet post-operatively was a beneficial gait modification, which may be associated with improved static and dynamic stability.

The positive influence of SDR on the central processing of different tasks including e.g. hand function and cognitive function has been described [55], [56] but not yet fully explained. This study shows that temporal parameters of variability can also be improved through SDR. One potential explanation is that the reduction of afferent input through SDR may ease the processing resources required by certain regions of the central cortex [16]. As regions that are known to play a major role in the variability of temporal stride intervals [23], the dorsolateral prefrontal cortex and the supplementary motor cortex (parts of the frontal lobe [16] that, together with other parts of the human brain, are responsible for task performance such as walking [57]), are likely to be affected by the greater afferent signalling input before SDR. In children with CP, the improved organisation and reduced levels of incoming information after SDR could have a positive influence on these areas and lead to improved task performance.

### Conclusions

SDR is known to improve task performance in children with CP. This study demonstrates for the first time, that temporal, but not spatial, parameters of variability are significantly affected after SDR treatment. This suggests that temporal variability of task performance is more susceptible to changes in afferent signalling, and therefore possibly more responsive to intervention therapies than spatial variability.
